# Regulation of perovskite oxides composition for the efficient electrocatalytic reactions

**DOI:** 10.1002/smo.20220005

**Published:** 2023-05-15

**Authors:** Jianyi Li, Yaxiong Yao, Li An, Shanshan Wu, Nan Zhang, Jing Jin, Rui Wang, Pinxian Xi

**Affiliations:** ^1^ State Key Laboratory of Applied Organic Chemistry Frontiers Science Center for Rare Isotopes College of Chemistry and Chemical Engineering Lanzhou University Lanzhou China

**Keywords:** defects, doping, exsolution, perovskite oxides, regulation composition

## Abstract

The benefits of perovskite oxides include their low cost, customizable composition, ordered atomic structure, and extremely flexible electronic structure. They are the ideal substitute for precious metal catalysts in various electrocatalytic reactions. However, the initial activity of perovskite oxides is often quite limited, which is extremely related to their crystal structure and electronic structure. In this regard, component regulation is the simplest and most effective strategy to increase their stability and catalytic activity. In this review, we briefly outline the recent progress in the modulating component of perovskite oxides to enhance their catalytic properties. The outline was categorized according to the sites in the ABO_3_‐type perovskite structure, including A‐site, B‐site, and O‐site regulation. Finally, potential research directions aimed at modulating of perovskite oxide constituents are discussed.

## Graphical abstract

The simplest and most effective strategy to enhance the catalytic activity of perovskite oxides is to regulate their composition. Here, we give a summary of recent advancements in the modification of perovskite oxides components to improve their catalytic characteristics. The outline is categorized based on the sites in ABO_3_‐type perovskite oxides, including A‐site, B‐site, and O‐site regulation.

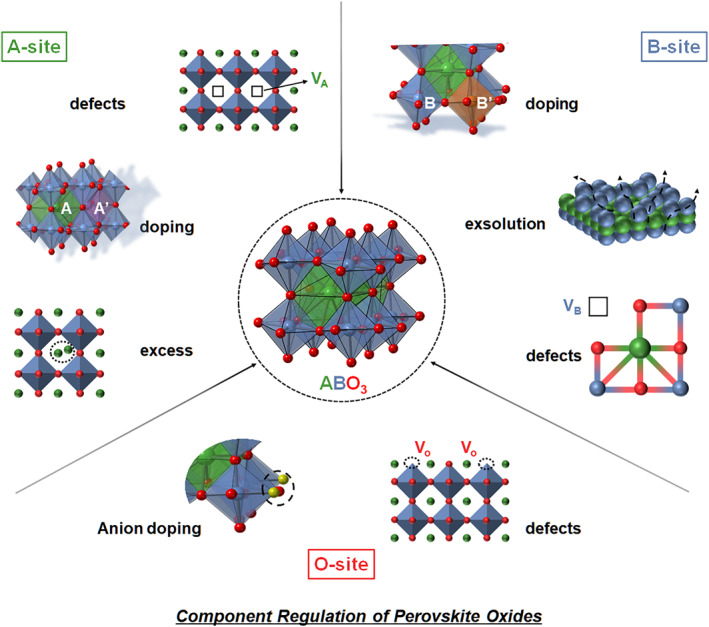

## 1 INTRODUCTION

**FIGURE 1:**
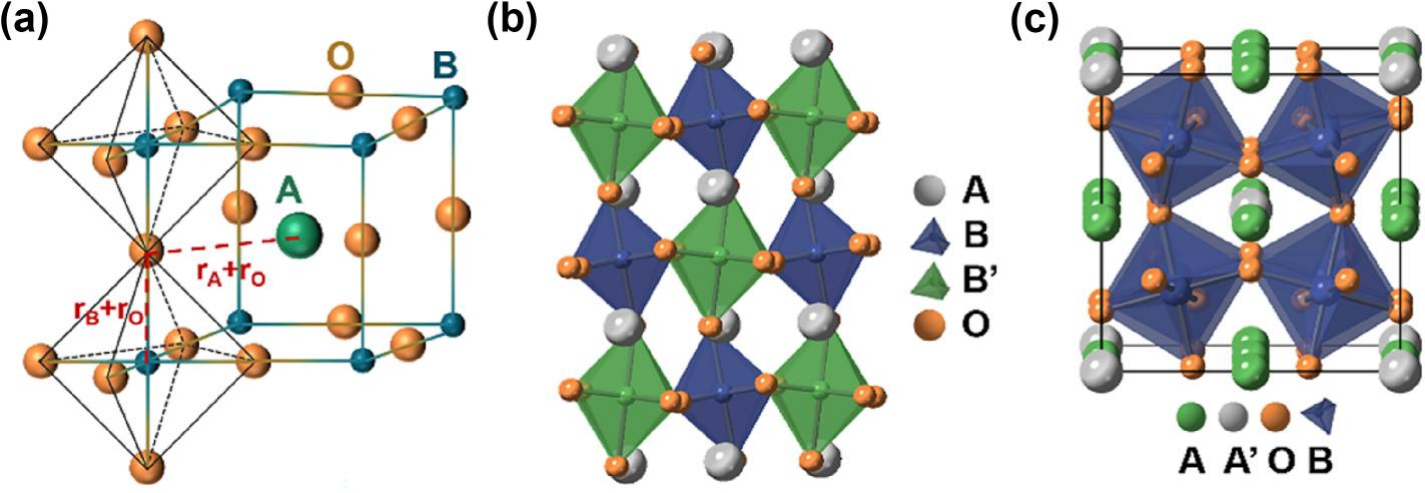
(a) ABO_3_ perovskite oxides' crystallographic structure. (b) B‐site order double perovskite oxides. (c) A‐site order quadruple perovskite oxides.

Perovskite is one of the most charming structures in the field of material science. The Russian mineralogist Gustav Ross discovered a mineral with the chemical formula CaTiO_3_ in 1839.^[1]^ Since then, materials with a similar structure to CaTiO_3_ have been classified as perovskite, and the general molecular formula is expressed as ABX_3_. Perovskite oxides can be described as ABO_3_, where A is an atom of alkaline earth metal (Ba, Sr, etc.), alkali metal (Li, Na, etc.) or rare earth metal (La, Pr, etc.), B is an atom of transition metal (Mn, Fe, etc.).[^2^, ^3^] Figure 1 depicts the A cation in twelve‐fold oxygen coordination and the B cation in six‐fold oxygen coordination. Additionally, the perovskite oxides family also includes double perovskites (A_2_B_2_O_6_), triple perovskites (A_3_B_3_O_9_), quadruple perovskites (A_4_B_4_O_12_), and Ruddlesden–Popper (RP) perovskites (A_n+1_B_n_O_3n+1_ (*n* = 1, 2, and 3)).[^4^, ^5^, ^6^]

Due to their low price, adjustable composition, ordered atomic arrangement and highly flexible electronic structure, perovskite oxides have undergone extensive research as the potential noble‐metal‐free electrocatalysts for metal‐air batteries,[^7^, ^8^] solid oxide fuel cells (SOFCs), and water splitting, including the oxygen reduction reaction (ORR),[^9^, ^10^, ^11^] the oxygen evolution reaction (OER),[^12^, ^13^, ^14^, ^15^] and the hydrogen evolution reaction (HER).[^16^, ^17^, ^18^] However, the large particle sizes, small surface areas, and poor electrical conductivities of perovskite catalysts often result in a limited amount of catalytic activity.^[19]^ In this regard, several strategies, such as composition engineering,[^20^, ^21^, ^22^] crystal facet control,[^23^, ^24^, ^25^] morphology control,[^26^, ^27^, ^28^] defect engineering,[^29^, ^30^] and hybridization,[^31^, ^32^, ^33^] have been developed to tune the physical and chemical properties of perovskite oxides for improving their catalytic activities.

Perovskite oxides exhibit remarkable flexibility in adjusting redox active sites and oxygen vacancies. The majority of elements in periodic table can be partially replaced by doping at the A‐ or/and B‐ or O‐ sites.^[34]^ The wide range of composition engineering options is determined by the great flexibility in composition, which in turn affects the physicochemical and catalytic properties. Consequently, one of the most facile and efficient methods to optimize the catalytic capabilities of perovskite oxides is by composition regulation.

**FIGURE 2:**
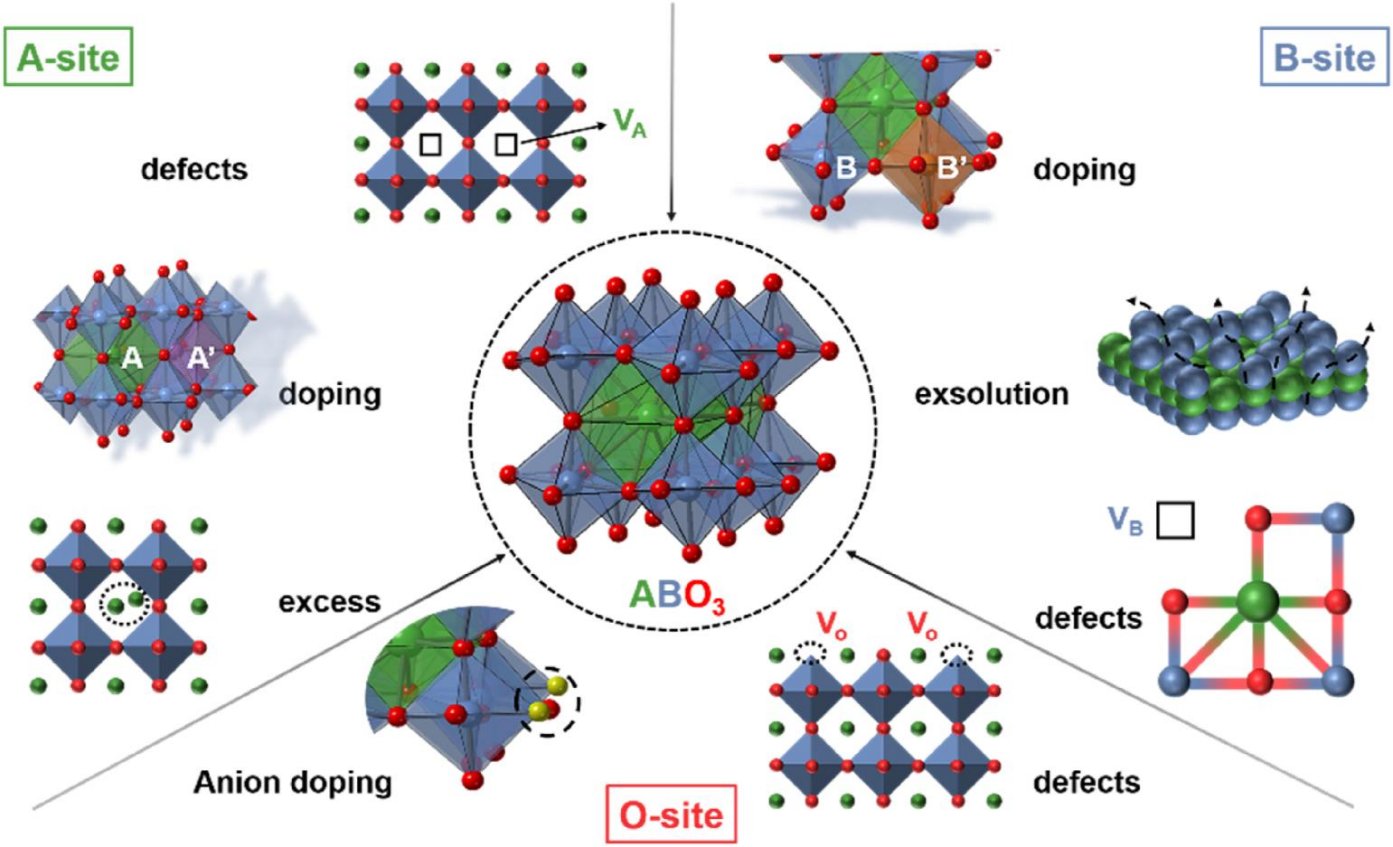
Diagram of the A‐site, B‐site, and O‐site regulation of ABO_3_‐type perovskite oxides.

There are many reviews focusing on perovskite electrocatalysts in terms of design principles, synthetic methods, and practical applications.[^19^, ^35^, ^36^, ^37^] Composition regulation strategies, as an essential subset of optimizing approaches, are rarely systematically and comprehensively reported. In this minireview, we have discussed the most recent development in composition regulation strategies that are concerned by reviewing perovskite oxide electrocatalysts from the perspective of different doping sites. The regulation of A site, B site, O site, and the hybridization with other materials is summarized (Figure 2). In the meantime, we have discussed how these perovskite oxides are used in diverse electrocatalysis applications. Finally, the remaining difficulties and future outlooks are described. Through the review, we hope to gain a thorough understanding of perovskite oxide electrocatalysts and offer guidelines for the reasonable design of highly efficient catalysts based on composition regulation.

## 2 REGULATION OF A‐SITE

During the electrocatalytic process, we usually consider that the A‐site metals are not participate in catalysis directly.[^37^, ^38^, ^39^, ^40^] However, it can indirectly influence the valence state of B‐site atoms, electronic conductivity, oxygen vacancies, and electrochemically active area in order to accomplish the goal of regulating the catalytic activity.[^39^, ^41^, ^42^] Based on this, researchers have investigated the modulation of A‐site to increase the electrocatalytic performance of perovskite oxides for different applications. The general formula of (A_1‐x_A'_x_)BO_3_/A_1‐x_BO_3_/A_1+x_BO_3_ is obtained by developing and inventing different techniques of A‐site cation, including doping, defect, and excess at A‐site of perovskite oxides.

### 2.1 A‐site doping

The oxidation states of A‐site metal cation are generally fixed, and it is hardly to change by the redox reaction in the electrocatalytic process.[^36^, ^43^] According to the apparent oxidation states of the doped atoms, we categorized and compiled the impacts of A‐site doping on the electrocatalytic activities in perovskite oxides, including isovalent metal doping and heterovalent metal doping.

#### 2.1.1 A‐site doping with an isovalent metal

**FIGURE 3:**
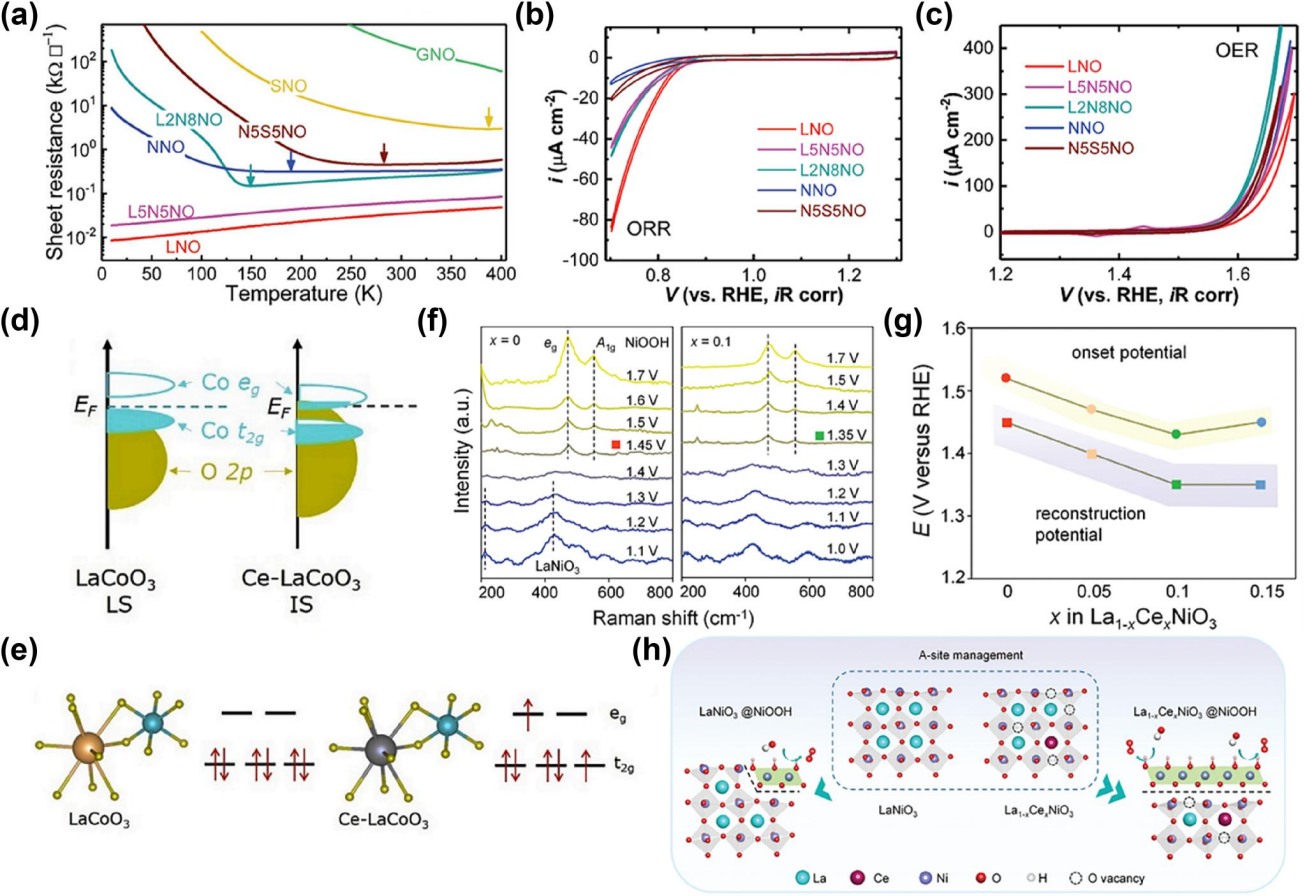
(a) Sheet resistance versus temperature on warming for RNiO_3_ films with different A‐site elements on STO substrates. The MIT temperature (colored arrows) are defined as the temperatures of the upturn in the sheet resistance versus temperature plots. Electrochemical results of La‐ and Nd‐contained nickelate films for ORR (b) and OER (c).^[44]^ Copyright (2018) Wiley. (d) Diagram of Co 3d‐O 2p overlap for LaCoO_3_/Ce‐doped LaCoO_3_. (e) Diagram of the orbital splitting of Co 3d in LaCoO_3_/Ce‐doped LaCoO_3_.^[46]^ Copyright (2020) Elsevier. (f) In‐situ Raman spectra of La_1−x_Ce_x_NiO_3_ (*x* = 0, 0.1) over the OER process (1.0–1.7 V vs. reversible hydrogen electrode). (g) The relationship between the onset potential for the OER and the surface reconstruction potential. (h) Diagram of surface reconstruction of La_1−x_Ce_x_NiO_3_ by the A‐site management of the Ce doping strategy.^[47]^ Copyright (2021) Wiley. OER, oxygen evolution reaction; ORR, oxygen reduction reaction; STO, SrTiO_3_.

Among rare‐earth nickelates family (RNiO_3_, here abbreviated RNO), LaNiO_3_ is the only oxide that is metallic at all temperatures, while the others all show a metal‐to‐insulator transition (MIT) at high temperatures (Figure 3a).^[44]^ In general, the rotation, tilt, and distortion of the NiO_6_ octahedra can be regulated by isovalent doping of R ions in RNO, thus affecting its structural and physicochemical properties.^[45]^ In 2018, Du and coworkers investigate a collection of well‐defined, epitaxial perovskite nickelates RNO (R = La, La_0.5_Nd_0.5_, La_0.2_Nd_0.8_, Nd, Nd_0.5_Sm_0.5_, Sm, and Gd) thin films grown on SrTiO_3_ (STO) (001) substrates by pulsed laser deposition (PLD).^[44]^ Then, the ORR and OER activities have been examined in O_2_‐saturated 0.1 M potassium hydroxide (KOH) electrolyte. The correlation between physical properties and ORR/OER activities was verified through extensive characterization. They revealed that the bond angle of Ni‐O‐Ni bends and the Ni–O hybridization decreases, as the radius of the A‐site ion decreases (change the A‐site element from La to Gd), resulting in the activity of ORR decreased significantly (Figure 3b). More importantly, the OER activity increased with the doping of Nd into LaNiO_3_ because of the energy decreased of the oxygen vacancies formation and a partial reduction of Ni^3+^ to Ni^2+^, which is maximized at La_0.2_Nd_0.8_NiO_3_ (L_2_N_8_NO) (Figure 3c). This study emphasized the importance of fine‐tuning A‐site elements as a practical method for effectively balancing ORR and OER activities.

The doping of rare earth metals in the A‐site of perovskite oxide also contributes to optimize the electronic structure, regulate the e_g_ electron filling, and increase the intrinsic conductivity.^[37]^ Ce doping is often considered to be a good accelerator to improve bifunctional activities. For example, Gao's group reported a Ce‐doped LaCoO_3_ perovskite oxide as an excellent bifunctional OER/ORR electrocatalyst.^[46]^ A combination of experimental and theoretical researches indicates that the introduction of Ce causes the spin state of the Co from the low‐spin state (LS) to the intermediate‐spin state (IS) and also results in the enlarged Co 3d‐O 2p covalency (Figure 3d,e). Herein lies the key to significantly enhancing the assembled rechargeable aqueous Zn‐air battery's performance.

The A‐site management strategies can also be used for activating the dynamic structure evolution. By using in‐situ electrochemical Raman spectra, Zhang et al. demonstrated that low‐concentration Ce doping promotes the self‐reconstruction of the perovskite LaNiO_3_ surface into the highly active NiOOH phase (Figure 3f).^[47]^ La_0.9_Ce_0.1_NiO_3_ provides a thirty‐two‐fold mass activity improvement with the overpotential of 270 mV at 10 mA cm^−2^ compared with LaNiO_3_ for the OER performance (Figure 3g). Through the engineered A‐site as a bridge, there is a practical route for perovskite oxide to prompt the B‐site element‐based active phase dynamic reconstruction (Figure 3h). The effects of doping the A‐site on the B‐site's properties and the enhancement of catalytic performance were substantiated by those experimental results.

#### 2.1.2 A‐site doping with a heterovalent metal

The doping with a heterovalent metal will cause charge imbalance in the whole perovskite oxide.[^37^, ^40^] According to the principle of electroneutrality, the oxidation state of B‐site active centers as well as the generation of oxygen vacancies can be regulated by such imbalance. These structural changes are effective factors to enhance the electrocatalytic activity and stability.

**FIGURE 4:**
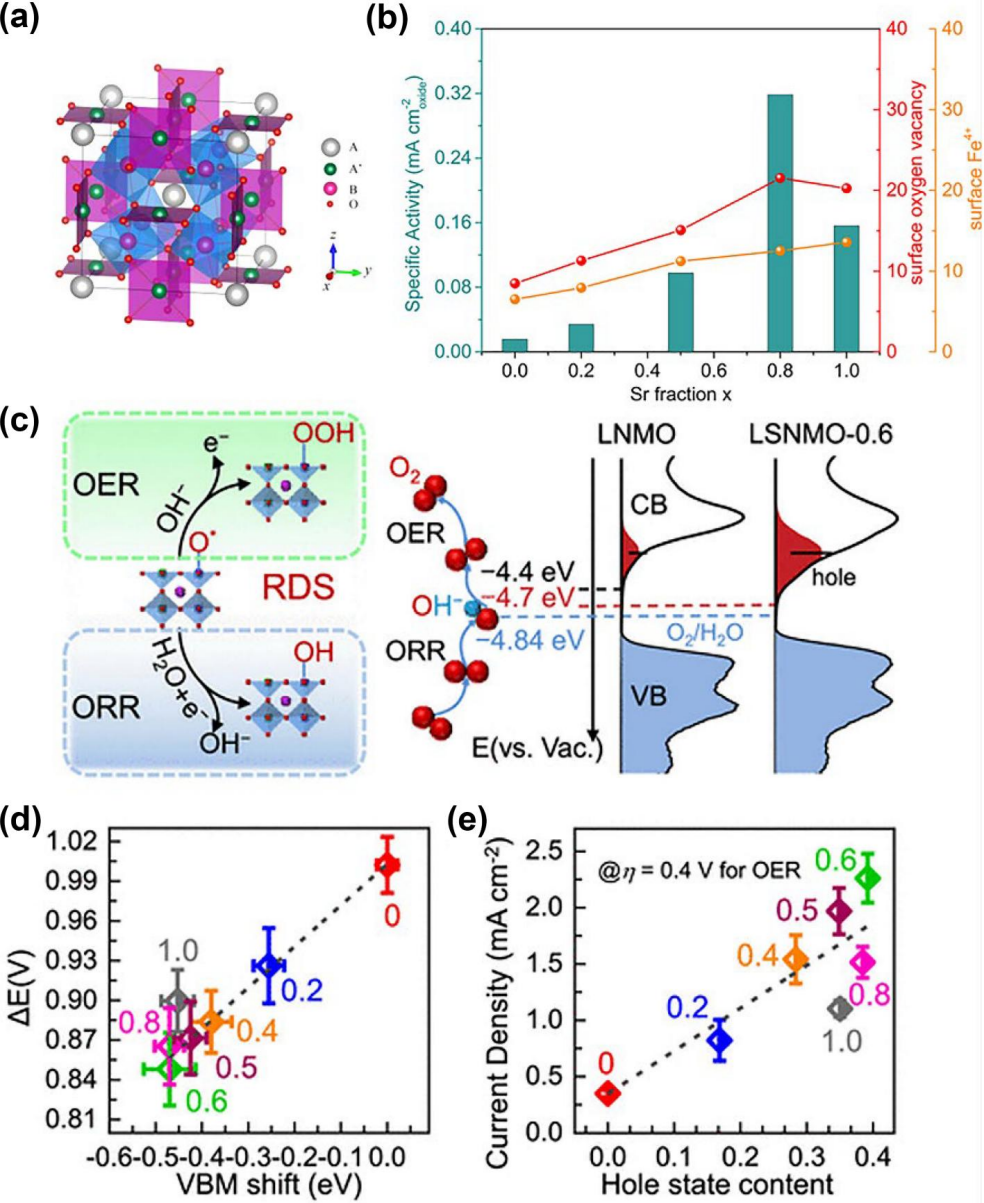
(a) Crystallographic structure of A‐site‐order perovskite AA'_3_B_4_O_12_.^[48]^ Copyright (2013) American Chemical Society. (b) Relationship between certain activities, surface oxygen vacancies, and surface Fe oxidation states for the LSF‐x (*x* = 0, 0.2, 0.5, 0.8, and 1) samples.^[50]^ Copyright (2018) American Chemical Society. (c) Left: rate‐determining step of the OER (O‐O bond formation in OOH^−^ adsorbate) and ORR (OH^−^ regeneration on transition metal site). Right: diagram for the electronic structure near Fermi leve at pH = 14; the conduction band minimum level for La_2_NiMnO_6_ is at −4.4 eV and the redox level for O_2_/H_2_O is located at −4.84 eV. (d) ΔE values as a function of value band maximum shift extracted. (e) Current densities at an overpotential of 0.4 V for the OER as a function of hole‐state content.^[51]^ Copyright (2021) American Chemical Society. OER, oxygen evolution reaction; ORR, oxygen reduction reaction.

In the A‐site ordered perovskite AA'_3_B_4_O_12_, the A‐ and A′‐site cations are arranged at the original twelve‐fold coordination A‐site in simple ABO_3_ with an additional valence transition metal ion at A′ site (Figure 4a).^[48]^ The valence variations of A‐site ions will lead to the synchronous change of the interaction between A'−A′ and A'−B as well as B‐B, which will also bring about rich physicochemical properties.^[49]^ Shimakawa and his coworkers successfully synthesized a series of AMn_3_V_4_O_12_ (A = Na^+^, Ca^2+^, and La^3+^) by a high‐pressure synthesis strategy.^[48]^ When the A‐site metal changing from Na^+^ to Ca^2+^ and Ca^2+^ to La^3+^, the electrons will first dope to the A′‐site Mn, changing Na^+^Mn^2.33+^_3_V^4+^_4_O_12_ into Ca^2+^Mn^2+^_3_V^4+^_4_O_12_, and then dope to the B‐site V, producing La^3+^Mn^2+^_3_V^3.75+^_4_O_12_. This demonstrated an unexpected site‐selective doping effect and associated changes in the characteristics, which is a new idea to effectively regulate the activity of perovskite oxides. To explore the influence of A‐site Sr doping on catalytic OER performance, Shao et al. synthesized a set of La_1−x_Sr_x_FeO_3−δ_ (*x* = 0, 0.2, 0.5, 0.8, 1) using the sol‐gel method.^[50]^ They found that Sr incorporation was accompanied by oxygen vacancy and the increase of Fe^4+^ (Figure 4b). The presence of Fe^4+^ is conducive to the adsorption of the active intermediate OH* and the catalytic activity of OER is improved. However, too much Fe^4+^ will hinder the desorption of OH* and reduce the catalytic activity. La_0.2_Sr_0.8_FeO_3−δ_ has the best performance with 0.37 V overpotential at a 10 mA cm^−2^. The incorporation of Sr in the double perovskite oxides La_2‐x_Sr_x_NiMnO_6_ (0 ≤ *x* ≤ 1.0) also has a similar improvement effect, which was confirmed by the report of the Zhang team.^[51]^ In addition to inducing the production of Ni^3+^, the valence band center is also shifted upward, and the hybridization of O 2p with the Mn 3d and Ni 3d orbitals is increased (Figure 4c,d). In addition, the Ni^3+^ also causes a rise in the density of unoccupied hole states, which effectively reduces the Schottky barrier transfer of electrons associated with OER from 0.44 to 0.12 eV, thus facilitating OER kinetics (Figure 4e). Bi doping is also commonly used to tune the electronic structure and surface acid‐base chemistry. For instance, Shao‐Horn et al. employed the inductive effect with more electronegative/Lewis acidic Bi ions into SrCoO_3_, which results in marked increase of the intrinsic OER catalytic activity.^[52]^

In short, A‐site doping (primarily including Bi, Sr, Na, and rare earth metals) is a successful strategy for improving the stabilities and catalytic activity of perovskite oxides.

### 2.2 A‐site cation defects

**FIGURE 5:**
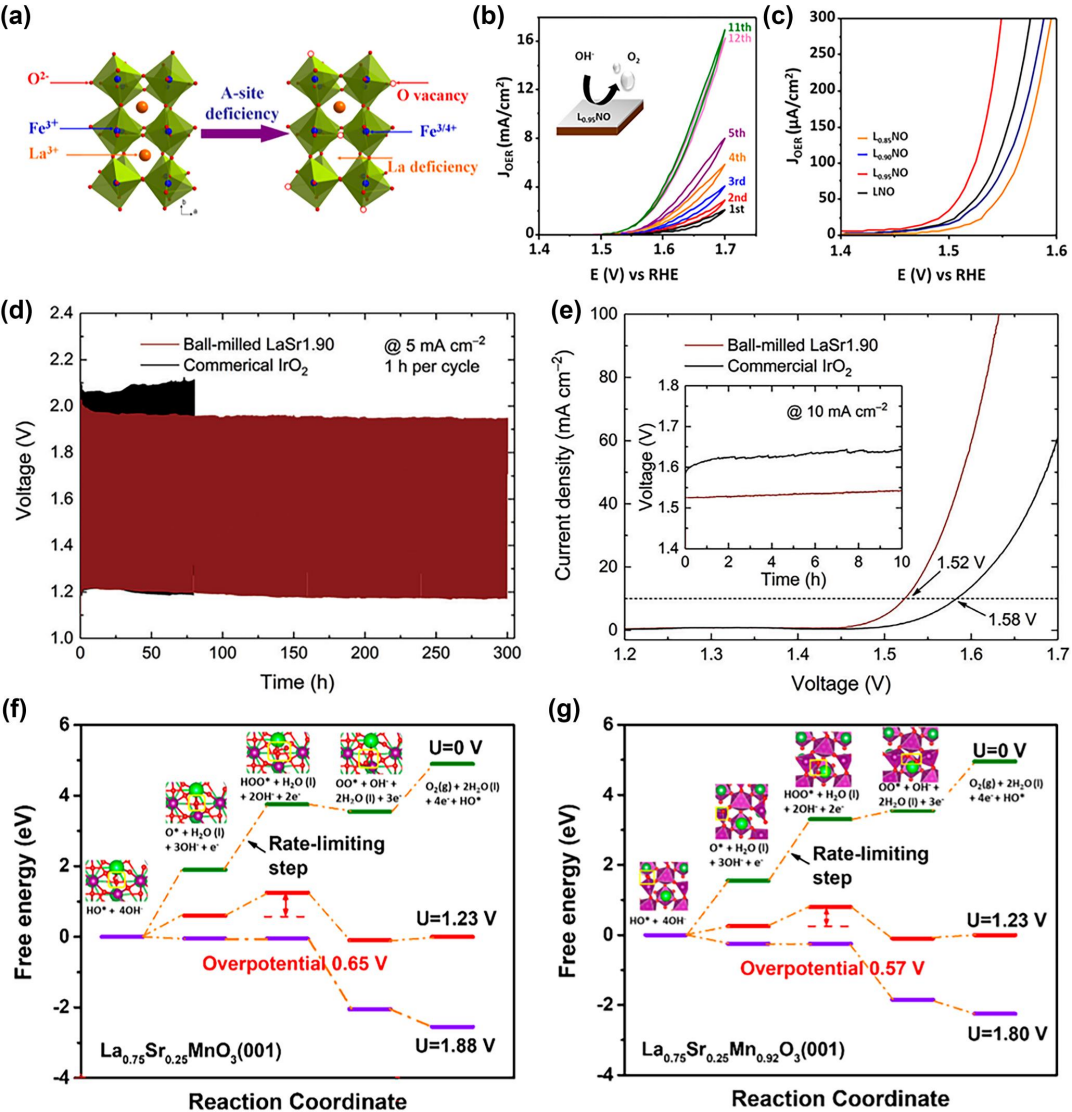
(a) Diagram of oxygen vacancy formation and Fe^4+^ in A‐site‐deficient La_1−x_FeO_3‐δ_ perovskites.^[57]^ Copyright (2016) American Chemical Society. (b) CV curves of L_0.95_NO electrode. (c) Linear sweep voltammetry curves of L_0.85_NO, L_0.90_NO, L_0.95_NO, and LNO films (NGO substrates).^[58]^ Copyright (2020) American Chemical Society. (d) Galvanostatic charge/discharge profiles of Zn–air batteries using ball‐milled LaSr_1.90_ and the commercial IrO_2_ catalysts. (e) Polarization curves of water electrolyzers using the ball‐milled LaSr_1.90_ and commercial IrO_2_. Anode catalyst and commercial 20 wt% Pt/C as a cathode catalyst. The inset shows the chronopotentiometry curves at 10 mA cm^−2^.^[59]^ Copyright (2022) Wiley. Free energy diagram of a four‐step OER mechanism on the (f) La_0.75_Sr_0.25_MnO_3_ and (g) La_0.75_Sr_0.25_Mn_0.92_O_3_ (001) surface in an alkali medium. In all diagrams, purple ball: Mn, fluorescent green ball: Sr, green ball: La, red ball: O, white ball: H.^[62]^ Copyright (2019) American Chemical Society. CV, cyclic voltammetry; OER, oxygen evolution reaction.

The ideal ABO_3_ perovskite structure is known to contain equal amounts of cations at the A sites and B sites (cation ratio A/B = 1). But in fact, when the A/B ratio deviates within a small reasonable range, the perovskite crystal structure can still exist stably.[^53^, ^54^, ^55^] It has been reported that the construction of A‐site cation deficiency is also an effective method to improve the intrinsic activity of perovskite oxides.^[56]^ For instance, Shao's team developed a straightforward and affordable procedure for synthesizing A‐site cation‐deficient La_1−x_FeO_3‐δ_ (*x* = 0.02, 0.05, 0.1) (Figure 5a), which significantly enhances the electrocatalytic activity of LaFeO_3_.^[57]^ Among the A‐site cation‐deficient perovskites reported, La_0.95_FeO_3‐δ_ demonstrated the best activity as the bifunctionality. They credited the formation of surface oxygen vacancies and the presence of a little quantity of Fe^4+^ species for this substantial improvement. It has been shown that excessive A‐site defects decrease OER activity by imposing different A‐site cation deficiencies on the LaNiO_3_ perovskite film (Figure 5b,c).^[58]^ The tilted NiO_6_ octahedron would fill the extra space created by La deficiency, causing the lattice shrinkage. On the other hand, the Ni‐O‐Ni bonding angle and charge transfer gap are altered by the A‐site cation deficiency in LaNiO_3_ films, leading to better oxygen evolution activities.

The A‐site defect strategy can also regulate the catalytic mechanism of perovskite oxides. An excellent platform for studying the contribution of the lattice oxygen‐mediated mechanism (LOM) has been provided to OER catalysis.^[59]^ The sol‐gel approach was adopted to introduce different levels of Sr deficiency in an La‐Sr‐Co‐Fe‐O series of LaSr_2−3z_, where 3*z* = 0.00, 0.05, 0.10, 0.15, and 0.20, denoted LaSr_2.00_, LaSr_1.95_, LaSr_1.90_, LaSr_1.85_, and LaSr_1.80_, respectively. The authors used soft X‐ray absorption spectroscopy (XAS) and X‐ray photoelectron spectroscopy (XPS) to determine that the Co/Fe oxidation state at site B remains unchanged. Therefore, it can be concluded that the compensation of charge imbalance caused by the introduction of Sr deficiency is achieved by the generation of oxygen vacancies, which follows the A‐site‐vacancy mechanism. This extra oxygen vacancy increases the oxygen diffusivity rate of the material. Electrochemical measurements show that modulation of the A‐site cation deficiency is a useful strategy for enhancing LOM‐based OER catalysis. They further assessed its practical applications in rechargeable Zn‐air batteries after being inspired by the remarkable OER activity of the optimized LaSr_1.90_ sample, which shows outstanding performance illustrated in Figure 5d,e.

All these cases prove that the strategy of A‐site cation deficiency can effectively improve the intrinsic activities of perovskite oxides.

### 2.3 A‐site cation excess

The physical and chemical characteristics of perovskite oxides, including A‐site deficiency and A‐site excess design, can be efficiently tuned using A‐site nonstoichiometry.^[53]^ Among them, A‐site excess is seldom seen in the perovskite field, especially in electrocatalysis.^[60]^ Shao et al. report a novel perovskite oxide, La excess in La_1.15_MnO_3+δ_, for a decreased average oxidation state of Mn cations and abundance oxygen vacancies.^[61]^ Compared to LaMnO_3+δ_, La_1.15_MnO_3+δ_ did show better catalytic performance in degrading Rhodamine B by activating peroxymonosulfate. The electronic structure of the majority of perovskite catalysts is only favorable for ORR or OER.[^9^, ^10^, ^11^, ^12^, ^13^, ^14^, ^15^] Specifically, Zhou and coworkers described a series of A‐site excessive perovskite oxides (La_0.8_Sr_0.2_)_1+x_MnO_3_ (*x* = 0.05, 0.1) via the polymer‐assisted chemical solution (PACS) method for both OER and ORR applications in alkaline media.^[62]^ (La_0.8_Sr_0.2_)_1.05_MnO_3_ has showed 21% higher diffusion‐controlled ORR current density and 87% higher OER current density at 0.8 V compared to LaSrMnO_3_. DFT calculations show that different A‐ and B‐ site stoichiometric ratios positively affect the OER kinetics without changing the reaction mechanism as shown in Figure 5f,g. Moreover, the upshift in the transition metal d‐band center and the oxygen p‐band center approaching the Fermi level are responsible for the improvement in the electrochemical performance of A‐site excessive (La_0.8_Sr_0.2_)_1+x_MnO_3_.

## 3 REGULATION OF B‐SITE

Different from the A‐site cation, numerous reports have demonstrated that B site is the active site that directly participates in catalytic reactions. By modifying the B site, we can introduce inert or active atoms directly into the catalytic system to improve the coupling efficiency of B'/B atoms and gain excellent physical and chemical properties. Thus, in order to effectively improve the catalytic efficiency, it is necessary to understand the regulation strategy of B site in perovskite oxides systematically and comprehensively.

### 3.1 B‐site doping with metals

Designing extremely effective electrocatalysts typically involves doping highly active noble metals into the B‐site of perovskite oxides. Recently, Xi et al. reported a small amount of Ir‐doped LaNiO_3_ at the atomic level by a liquid‐phase ion exchange strategy.^[63]^ With the optimal condition, LaNi_0.96_Ir_0.04_O_3_ exhibits an overpotential of 280 mV at 10 mA cm^−2^ in alkaline media. DFT calculations and XPS and XAS analyses demonstrated that the actual catalytic site switched from Ni to Ir. Combined with cyclic voltammetry (CV) characterization, the origin of the high OER activity was revealed by the strong covalent interaction between Ni‐O‐Ir. Furthermore, in‐situ differential electrochemical mass spectroscopy (DEMS) and in‐situ attenuated total reflection infrared (ATR‐IR) spectra were used to demonstrate that the incorporation of Ir inhibited the degree of involvement of lattice oxygen mechanism (LOM), slowed down the dissolution of Ni, and enhanced the stability of the catalyst.

**FIGURE 6:**
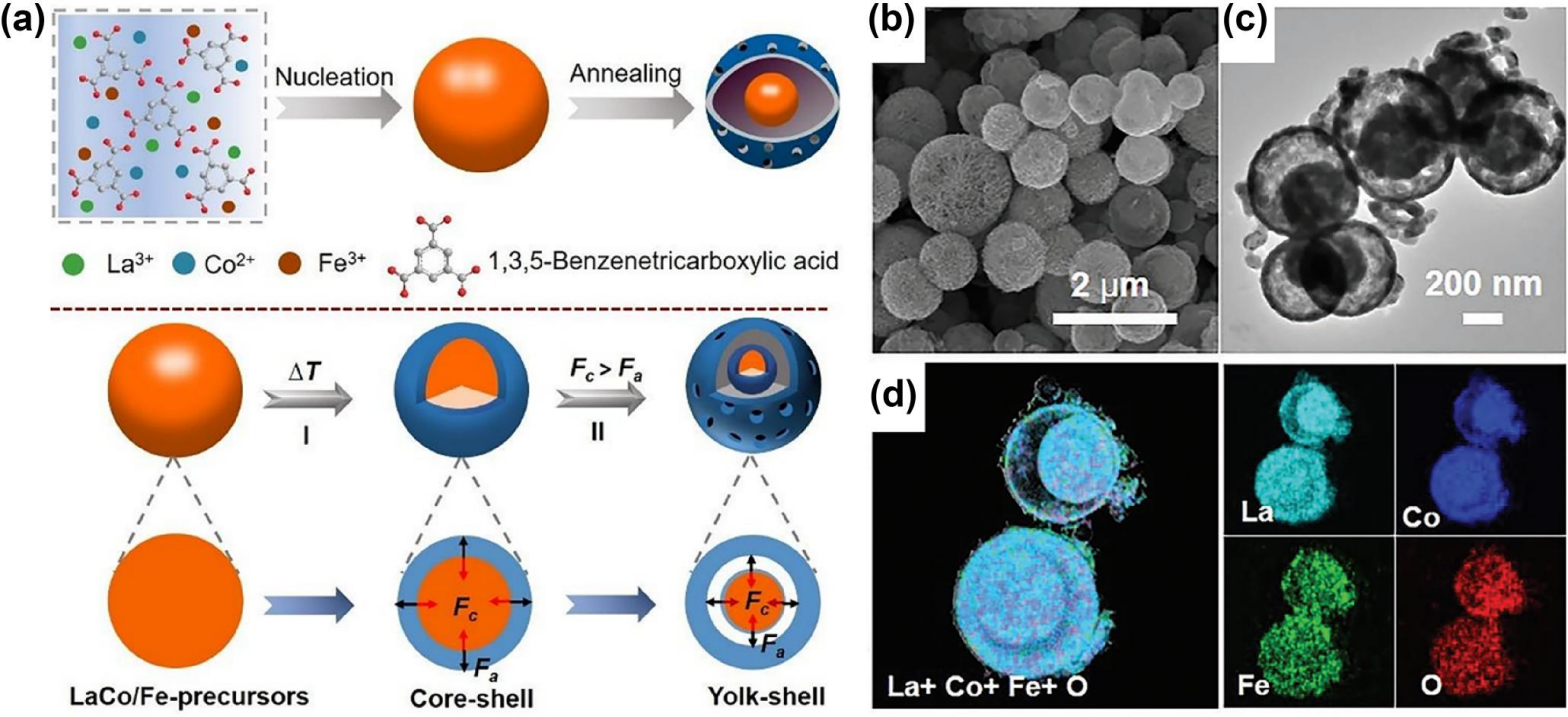
(a) Diagram for the preparation of LaCo_1‐x_Fe_x_O_3_ YSNs (*x* = 0.00, 0.10, 0.25, 0.50, 0.75 and 1.00). (b) SEM image, (c) TEM image, and (d) elemental mappings of LaCo_0.75_Fe_0.25_O_3_.^[64]^ Copyright (2022) Wiley. YSNs, yolk‐shell structures.

In general, non‐precious transition metals (such as Fe, Co, etc.) are more widely used as B‐site dopants due to their low cost and abundant earth resources. Recently, Xi and coworkers fabricated a series of Fe‐doped LaCoO_3_ perovskite microspheres possessing yolk‐shell structures (YSNs) by a ligand‐assisted method.^[64]^ The overall strategy for the fabrication of LaCo_1‐x_Fe_x_O_3_ YSNs is shown in Figure 6a. This synthetic approach is highly adaptable and can be applied to create precisely controlled compositions of various perovskite oxides. Owing to the benefits of the yolk‐shell architecture and the B‐site partial doping of perovskite using Fe, LaCo_0.75_Fe_0.25_O_3_ (Figure 6b–d) displays an overpotential of 310 mV at 10 mA cm^−2^ for OER. To further understand the improved OER performances, DFT calculations are carried out and demonstrated that 3D orbitals of Fe exhibit a pinning effect on 3D orbitals of Co to maintain a stable valence state of Co sites at the low overpotential. According to in‐situ Raman spectroscopy, the B‐site doping promotes the pre‐oxidation of Co sites and triggers the surface reconstruction into active Co oxyhydroxides at lower applied voltage. Besides, the Zn–air batteries assembled with the optimal material display a high open‐circuit potential of 1.47 V, superior energy density of 905 Wh kg^−1^_Zn_, good mechanical flexibility and stability in a large temperature range. This work provides an effective strategy and broad applications for designing perovskite‐based electrocatalysts.

**FIGURE 7:**
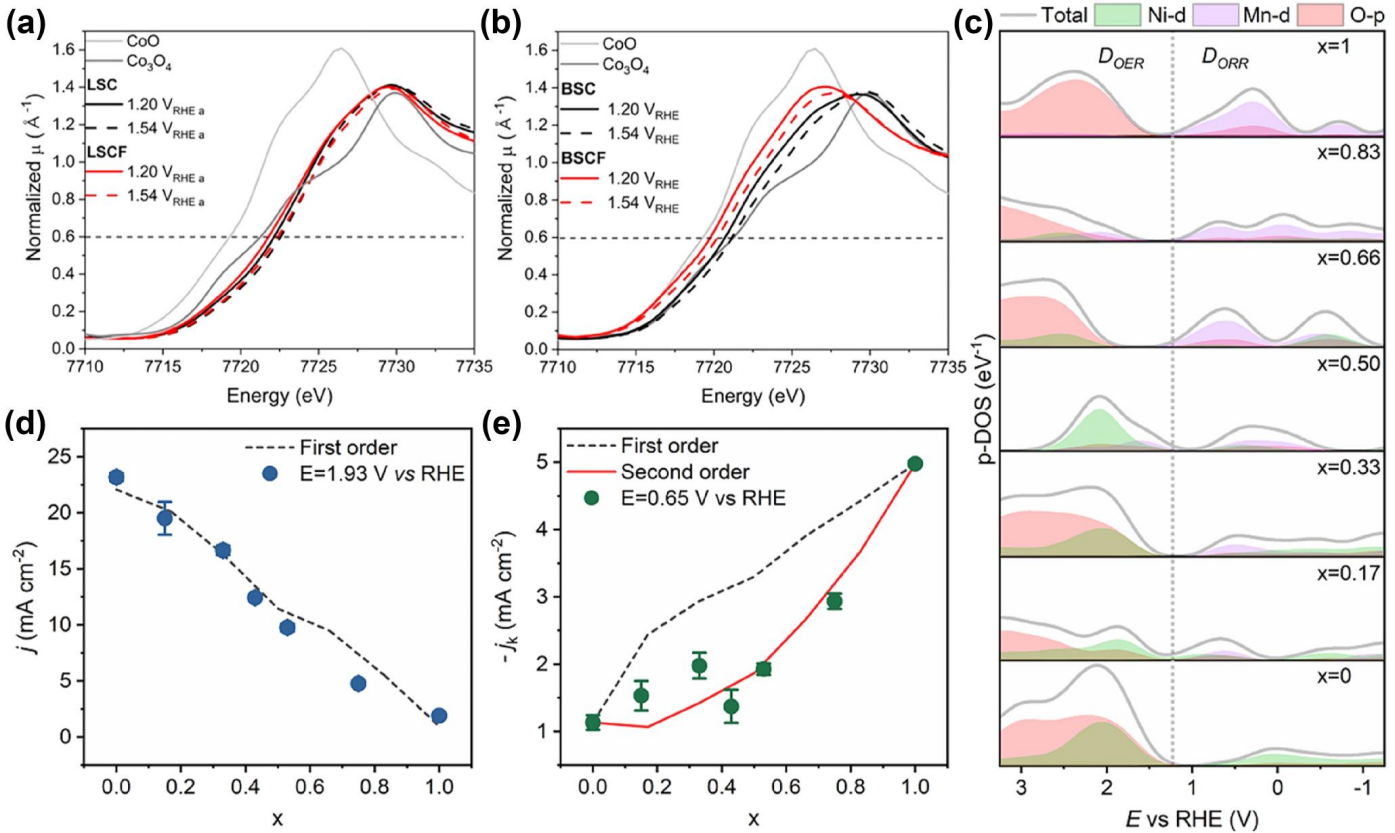
Comparison of Co K‐edge XANES spectra recorded at 1.2 and 1.54 V_RHE_ of (a) as‐prepared La_0.2_Sr_0.8_CoO_3‐δ_ and La_0.2_Sr_0.8_Co_0.8_Fe_0.2_O_3‐δ_; (b) as‐prepared Ba_0.5_Sr_0.5_CoO_3‐δ_ and Ba_0.5_Sr_0.5_Co_0.8_Fe_0.2_O_3‐δ_.^[66]^ Copyright (2019) American Chemical Society. (c) Elemental‐projected DOS in the RHE potential scale for the various LMNO compositions. The dotted line corresponds to 1.23 V versus RHE. (d) Composition dependence of the OER current density extracted for experimental data at 1.93 V versus RHE and from the first‐order independent active site model. (e) Composition dependence of the kinetically limited ORR current at 0.65 V versus RHE compared to the trends expected for a first‐ and second‐order independent active site model.^[67]^ Copyright (2022) American Chemical Society. BSCF, Ba_0.5_Sr_0.5_Co_0.8_Fe_0.2_O_3‐δ;_ DOS, density of states; OER, oxygen evolution reaction; ORR, oxygen reduction reaction; RHE, reversible hydrogen electrode.

According to Sabatier's principle, an ideal electrocatalyst should have a moderate surface oxygen interaction energy (not too strong, not too weak).^[65]^ In 2019, Schmidt et al. investigated the synergistic effect of Fe and Co at B site on the OER catalytic activity.^[66]^ Using ex‐situ and in‐situ XAS, the authors clarified that the addition of Fe made the Co oxidation state lower, inhibited the tendency of Co to generate oxidation by‐products, and promoted its self‐assembly into a highly catalytic active hydroxide layer (Figure 7a,b). The growth rate of Co valence after doping is higher during OER, indicating that the catalytic activity can be effectively increased through the doping regulation of Fe. For the first time, Fermin et al. determined the dominant role for the integrated density of states (DOS) of transition metals in OER and ORR reactions under operating conditions, which contributed to a deeper understanding of the importance of orbital occupancy.^[67]^ The analysis of pseudocapacitive responses in the potential region by systematic electrochemistry revealed that the Ni d‐orbitals evolved synchronically with the raise of Mn content **(**Figure 7c). Combined with DFT calculations, the results display a linear relationship between the kinetics and the DOS of Ni and Mn states in OER (Figure 7d), but a second‐order reaction with respect to the ORR (Figure 7e).

Naturally, we conclude that the doping regulation of the active metal at the B site is the simplest and most effective way to regulate the activities of the catalysts.

### 3.2 B‐site doping with non‐metals

**FIGURE 8:**
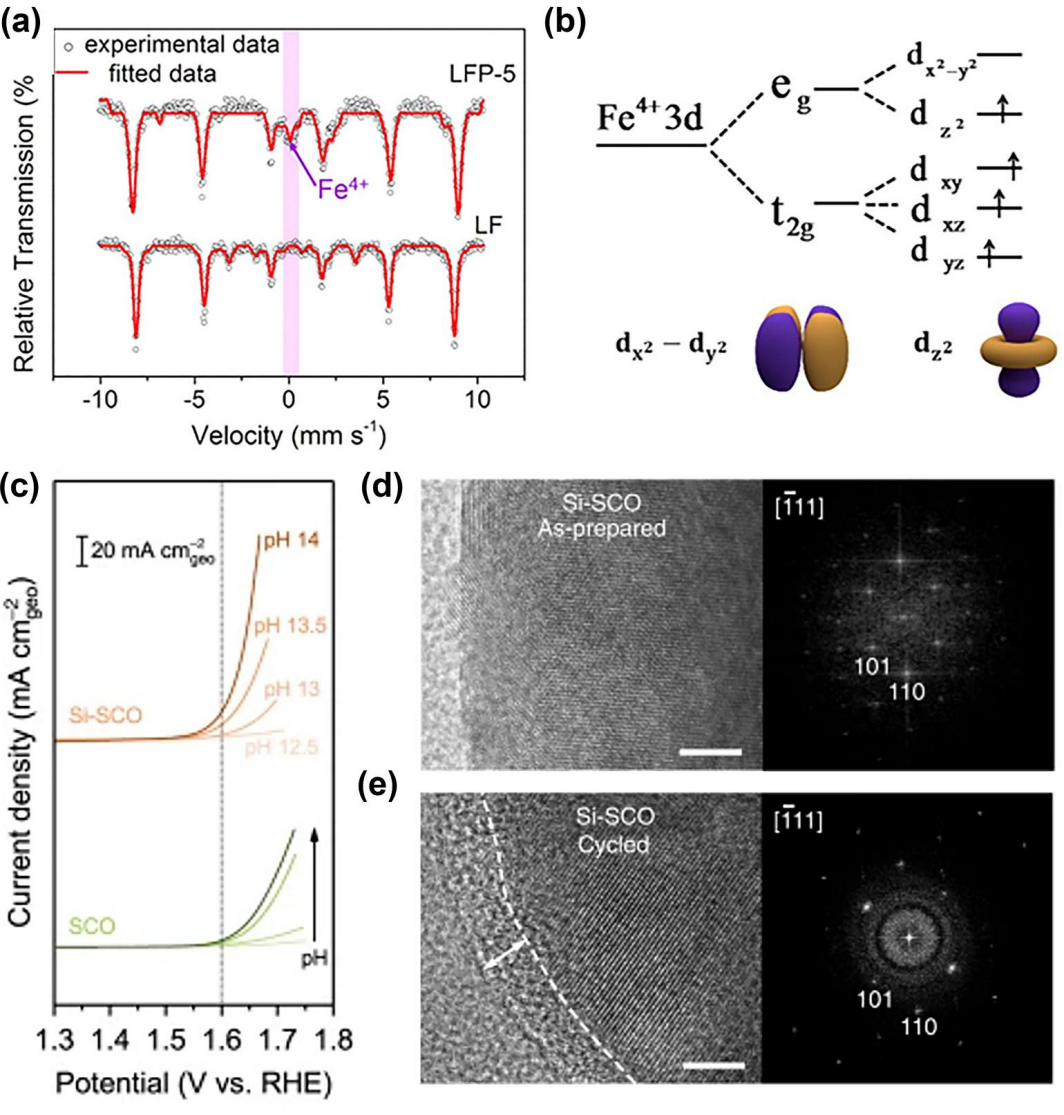
(a) ^57^Fe Mössbauer spectra of LF and LFP‐5 at 298 K. (b) Diagram of 3D orbit of Fe degeneration.^[69]^ Copyright (2018) Elsevier. (c) OER kinetic currents (mA cm^−2^_geo_) of SrCoO_3‐δ_ (SCO) and Si–SCO by varying pH in O_2_‐saturated KOH electrolytes. HRTEM images and the corresponding fast Fourier transformed patterns of (d) as‐prepared and (e) cycled (for 50 cycles) Si–SCO electrode.^[71]^ Copyright (2020) Nature Publishing Group. OER, oxygen evolution reaction.

As a supplement to the metal regulation strategy at B‐site, introducing non‐metallic ions is another efficient tactic to improve the activity and stability of catalysts.^[68]^ In 2018, Liu and coworkers selected LaFeO_3_ as the structural model and then studied the effect of introducing P for its bifunctional oxygen activity.^[69]^ As showed in X‐Ray diffraction (XRD), no structure or phase transition was observed although the synthesis temperature increased from 300°C to 600°C. The perovskite surface analysis indicated the introduced large amount of the O_2_^2−^/O^−^ species on the perovskite surfaces upon phosphorus doping. Beyond that, the trace amount of Fe^4+^ was observed (Figure 8a). The coexistence of Fe^4+^/Fe^3+^/Fe^2+^ led to the e_g_ electron filling factor being close to 1 (Figure 8b). Theoretical calculation showed that the introduction of P leads to the partial occupation of Fe 3d_yz_ orbital and the increase of pore states enhanced the electrophilicity of absorbed O and promoted the absorption of ‐OH at the catalytic active site. Remarkably, LFP‐5 (the material synthesized under 500°C) delivers a higher ORR performance and OER activity with the smallest onset potential 1.628 V_RHE_ and the largest electrocatalytic current density, relative to the pure LaFeO_3_.

Si doping has also been proved to effectively enhance the catalytic performance of perovskite oxides. Shao et al. reported Si‐doped OER catalysts SrFeO_3−δ_.^[70]^ The extremely effective SrFe_0.9_Si_0.1_O_3−δ_ was found to significantly increase the activity by a factor of about three. This elevated activity has been attributed to a structural transition from tetragonal to cubic symmetry, the latter with optimized cationic states, abundant oxygen vacancies, and fast charge transfer. In 2020, the authors demonstrated that Si‐incorporated could enhance the participation of lattice oxygen in another work.^[71]^ By tailoring the content of Si, the authors developed a variety of materials with various levels of oxygen vacancy and oxygen diffusion rates.

The surface amorphization observation and pH‐dependent OER kinetic studies show that the LOM mechanism is effective during the OER of SrCoO_3‐δ_ (SCO) and Si‐doped SCO (Figure 8c–e). It is worth noting that the intrinsic activity is improved up to an order of magnitude when the inclusion of Si into the SCO lattice, which was close to the activity of the typical example Ba_0.5_Sr_0.5_Co_0.8_Fe_0.2_O_3‐δ_. Besides, the oxygen diffusivity of Si‐doped perovskite is increased by about thirteen‐fold, which matches well with the ten‐fold enhancement of intrinsic OER activity, indicating that the observed increase is mainly the result of the enhancing the participation of lattice oxygen.

These findings showed that non‐metal element doping is a useful technique for enhancing the ion valence states and oxygen defect content of perovskite electrocatalysts, regulating the catalytic mechanism, and enhancing the activities.

### 3.3 B‐site cation defects

**FIGURE 9:**
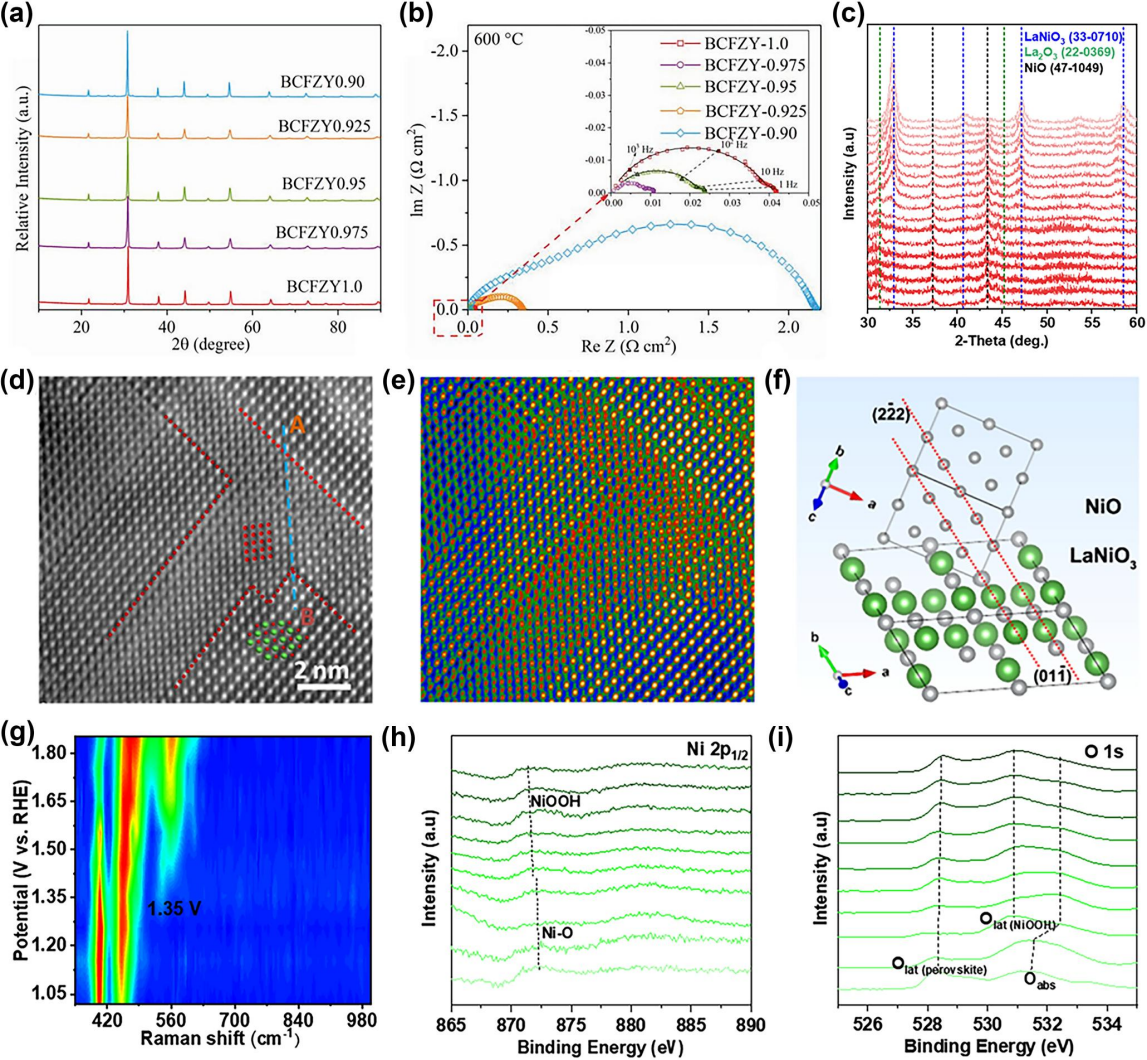
(a) X‐Ray diffraction (XRD) patterns of B(CFZY)x (*x* = 1.0, 0.975, 0.95, 0.925, and 0.90) samples sintered at 1000°C in air for 5 h. (b) Nyquist plots of the electrochemical impedance spectroscopy of B(CFZY)x (*x* = 1.0, 0.975, 0.95, 0.925, and 0.90) oxygen reduction electrodes obtained from the symmetric cells at 600°C in air.^[73]^ Copyright (2019) Wiley. (c) Ex‐situ XRD patterns on this sample (R = 0.8) were carried out from 400°C to 700°C and maintained at the desired temperature for 5 min under oxygen enrichment. (d) Atomic HAADF‐STEM image and (e) fast Fourier transformed‐filtered image of LaNiO_3_/NiO with a [011] orientation. (f) Crystallographic structure of the LaNiO_3_ and NiO proposed based on the HAADF‐STEM image. (g) Potential‐dependent operando Raman spectra of LaNiO_3_/NiO. Quasi in‐situ Ni 2p and O 1s XPS spectra for (h,i) LaNiO_3_/NiO at a specific potential from 1.02 to 1.85 V (vs. reversible hydrogen electrode).^[75]^ Copyright (2022) American Chemical Society.

Relevant studies have been published significantly less frequently than A‐site deficiency because the production of B‐site cation deficiency is less advantageous in terms of energy.^[53]^ In 2015, Liu et al. designed a novel perovskite oxide La(Co_0.55_Mn_0.45_)_0.99_O_3−δ_ (LCMO) via a hydrothermal method and heat treatment.^[72]^ LCMO showed the morphology of uniform nanorods and the 1% B‐site vacancy, which exhibits high intrinsic activities for ORR and OER. The ORR performance of perovskite oxides at reduced temperature could be improved by adjusting the surface properties and bulk diffusion properties.^[73]^ For example, Shao et al. introduced a slight B‐site cation deficiency into BaCo_0.4_Fe_0.4_Zr_0.1_Y_0.1_O_3−δ_ (BCFZY), namely BCFZY_0.975_ and BCFZY_0.95_, and the perovskite lattice structure was retained. By further increasing the nominal B‐site cation deficiency, the impurity phase appeared (Figure 9a). This suggested excessive B‐site cation deficiency may destroy the structural stability of perovskite. Further, BCFZY_0.975_ and BCFZY_0.95_ showed significantly improved activity for ORR (the area‐specific resistances of 0.011 and 0.024 Ω cm^2^ at 600°C), which are higher than that of the cation stoichiometric BCFZY (Figure 9b). This improvement is attributed to enhanced surface exchange and bulk diffusion kinetics due to increased oxygen mobility.

Notably, a viable strategy for improving the activity of perovskite oxides is to introduce a B‐site cation deficiency, but the level of deficiency shall be carefully controlled.

### 3.4 Exsolution from perovskites

One of the characteristics of perovskite oxides is that transition or noble metals can be doped to the B‐site of the lattice. Consequently, the perovskite lattice can serve as a reservoir for the dopant to be released after post‐processing or in a reduction reaction environment. Dopants move to the surface during exsolution and form stable components, resulting in highly active catalyst surfaces.[^53^, ^74^]

Some specific cations may exsolve in some nonstoichiometric perovskites; after heat treatment, an interface between the perovskite crystal and the exsolution content is created. Recently, Xi et al. constructed a unique interface between the A‐site deficiency LaNiO_3_ and foreign compounds NiO by deliberately controlling the composition proportion to tend the phase segregation in the parent perovskite.^[75]^ The phase transition and intermediate states of R = 0.8 samples (R is defined as the molar ratio of La to Ni) at different annealing temperatures were studied by XRD. As shown in Figure 9c, La_2_O_3_/NiO heterostructures were formed below 550°C. When the temperature was higher than 570°C, LaNiO_3_ was preferentially grown and La_2_O_3_/LaNiO_3_/NiO heterostructure was formed. At 700°C, La_2_O_3_ is completely consumed and NiO is first weakened and then enhanced. Finally, thermally stable LaNiO_3_/NiO heterogeneous materials were formed. High‐angle annular dark‐field scanning transmission electron microscopy (HAADF‐STEM) atomic resolution image is shown in Figure 9d,e. The interface is formed by {100} NiO nanopores embedded in the {100} LaNiO_3_ matrix (Figure 9f). DFT calculations showed that the formation energies of all three heterostructures are negative, which are consistent with the increased formation temperature during synthesis. The operando Raman spectroscopy was used and revealed that the LaNiO_3_/NiO heterostructure could greatly promote the production of an active self‐constructed surface NiOOH layer during the OER process, which boosted the OER performance (Figure 9g). The quasi in‐situ XPS also demonstrated consistent results (Figure 9h,i). The LaNiO_3_/NiO heterostructure had the expected good OER activity with an overpotential of 334 mV.

Similarly, Shao's group reported an efficient basic HER hybridization catalyst constructed based on a one‐step exsolution process of growing metal nanoparticles (NPs) in‐situ from perovskite oxide hosts.^[76]^ La_0.4_Sr_0.4_Ti_0.9_Ni_0.1_O_3‐δ_ (LSTN) precursors were firstly synthesized using sol‐gel strategy followed by calcining at 900°C for 10 h under 10% H_2_/90% Ar atmosphere to obtain the Ni NPs/La_0.4_Sr_0.4_Ti_0.9_Ni_0.1_O_3‐δ_ (e‐LSTN). The valence state of surface elements was analyzed by XPS, the outcomes unmistakably showed that the exsolution process only causes the active Ni in LSTN to be reduced and has no impact on other elements. LST promoted the dissociation of water and the production of hydrogen intermediates (H_ads_), while Ni NPs promoted the adsorption of hydrogen intermediates and the production of H_2_, and this synergistic effect made its HER activity much higher than that of the physically mixed LST/Ni. In addition, extensive experiments were carried out to demonstrate that the catalytic activity of the catalyst can be tuned according to the type of metal/alloy and the acidity of the perovskite substrate. However, it has been still very difficult to observe ion exsolution processes with sufficiently high spatial and temporal resolutions at the atomic scale.

**FIGURE 10:**
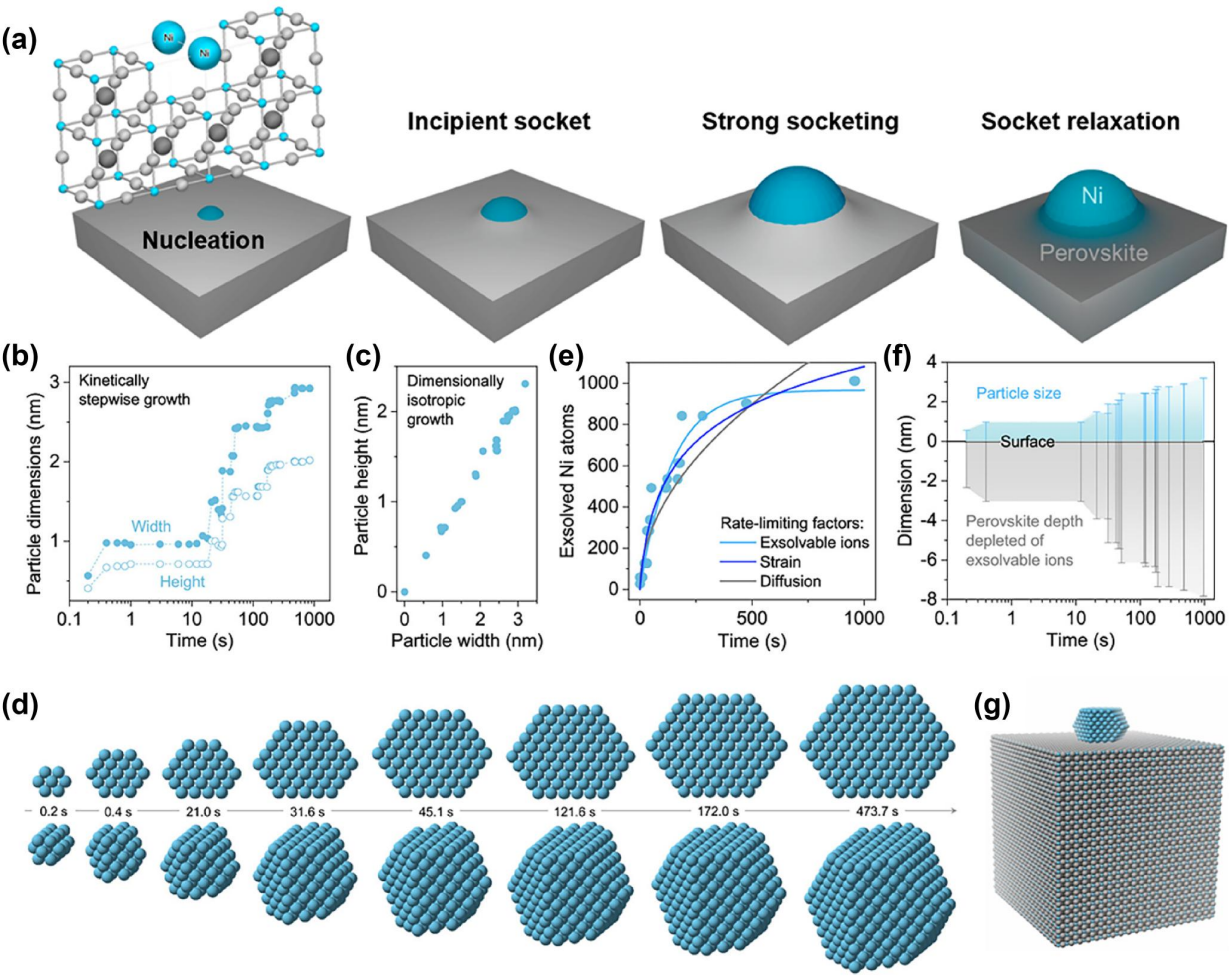
(a) Diagram of particle‐socket genesis during exsolution. (b) Diagram of the width and height of the particle as a function of time. (c) Diagram of the particle width versus height. (d) 2D and 3D models of the time evolution of a particle during exsolution. (e) Diagram of the number of Ni metal atoms contained within the particle as a function time together with various models describing the rate‐limiting processes that govern particle growth. (f) Diagram of the average particle size and corresponding depth within the perovskite that has been depleted of Ni^2+^ ions. (g) 3D model of a particle in final stages of growth and the corresponding perovskite volume required to form it.^[77]^ Copyright (2019) American Chemical Society.

In 2019, Tsampas et al. captured the exsolution dynamics of individual nanoparticles with ultra‐high spatial and temporal resolutions using the most recent environmental transmission microscopy technology.^[77]^ La_0.43_Ca_0.37_Ni_0.06_Ti_0.94_O_3_ and La_0.8_Ce_0.1_Ni_0.4_Ti_0.6_O_3_ were selected as the study samples with the former having a low exsolvable Ni content relative to the latter. In‐depth analysis of the TEM showed that the metal particles grow epitaxially and isotropically after dissolution, and the specific position does not move with time but maintains the initial nucleation position, while the perovskite lattice in the vicinity of the particles rises simultaneously. As the particles grow, the perovskite lattice exhibits lateral expansion, forming a “volcanic”‐shaped socket. Over time, the volcano‐like nanostructures eventually form a relaxed but still stable exsolution interface in order to reduce surface tension (Figure 10a). Furthermore, the authors carried out exsolution experiments in the ETEM vacuum environment to slow down the particle growth process, indicating the kinetics of particle nucleation and growth during exsolution by calculating the width and height of the particles (Figure 10b–g). The growth process is not homogeneous, but increases in a discrete and gradual manner, with alternating intervals and sudden growth periods. This proved that exsolution is a highly controlled process until individual particles are generated.

## 4 REGULATION OF O‐SITE

In the electrocatalytic process, the O‐site is crucial, which can not only participate the catalytic procedure, but also has a series of influence to the material properties, such as electronic conductivity, spin state of B‐site, and so on.[^5^, ^53^, ^78^] Therefore, modulation of O‐site to get better catalytic performance has been widely investigated by researchers.

### 4.1 O‐site doping

The O‐site can be doped by many elements with similar radius, such as N, S, P, and halogen.[^79^, ^80^, ^81^] The doping of O‐site mainly changes the electronic conductivity and the spin state of B‐site.^[81b]^

**FIGURE 11:**
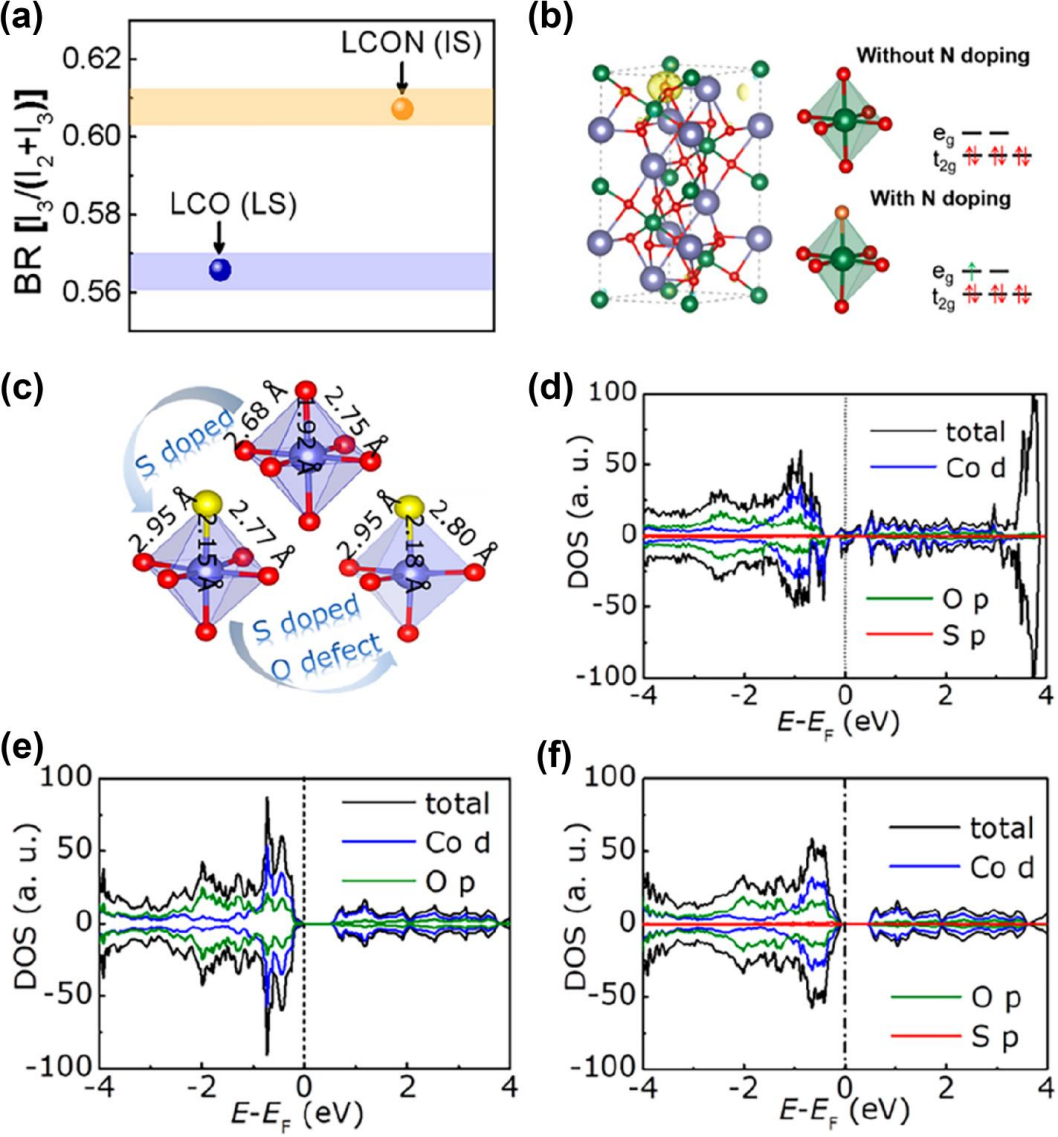
(a) Branching ratio of LaCoO_3_ (LCO) and LCON derived from the Co‐L edge. (b) Spin density distribution and electronic configuration of LCO and N‐doped LCO.^[83]^ Copyright (2019) American Chemical Society. (c) Corresponding optimized atomic structures. DOS diagram of (d) S‐doped LCO with an oxygen defect, (e) LCO and (f) S‐doped LCO.^[84]^ Copyright (2020) American Chemical Society.

The electron configuration of e_g_ is a significant descriptor for OER catalytic activity of perovskite oxides.^[82]^ The absorption of oxygen‐containing groups on the cations in octahedral sites can be improved by the electron configuration of e_g_ = 1. LaCoO_3_ has a low spin state (LS, e_g_ = 0), which can turn into intermediate spin state (IS, eg = 1) to have better catalytic performance. To provide an extra electron to the d orbital of Co cations, N dopants can share orbitals with cations. This modifies the spin states of LaCoO_3_, which improve OER and ORR capabilities. Therefore, Gao and coworkers investigated a round of contents of N dopants by annealing LaCoO_3_ in NH_3_ for 0.5, 1, and 1.5 h.^[83]^ Finally, N was successfully doped to replace the O atom, resulting in the transfer of the spin state of the Co cation in LaCoO_3_ (LCO) from LS to IS **(**Figure 11a,b). With a current density of 50 mA/cm^2^, the optimum OER overpotential falls from 1.94 to 1.69 V, and the ORR limiting current density rises from 4.01 to 5.78 mA/cm^2^.

Besides N‐doped LaCoO_3_ (LCON), Gao also proved that S dopant in LaCoO_3_ has a similar effect to the electrocatalytic activity.^[84]^ In 2020, Gao and coworkers prepared S‐doped LaCoO_3_ by sulfurization of sulfur powder in an argon atmosphere at 400°C for 2 h. According to DOS, the authors elaborated that the introduction of S facilitates the enhancement of LCO conductivity (Figure 11e,f). The oxygen defect also played an important role in promoting the occupation of electronic states at the Fermi energy level (Figure 11d). Meanwhile, the spin state transferred from LS to IS after S doping, which is proved by EELS and magnetic properties. The improvement of Electrocatalytic activities for both the OER (an overpotential = 364 mV at 10 mA/cm^2^) and ORR (limiting current density = 4.5 mA/cm^2^ at 0.2 V) was observed. Meanwhile, S‐doped LaNiO_3_ and LaFeO_3_ were also synthesized, respectively, with better bifunctional catalysis and high stability. They demonstrated how sulfur doping in oxide perovskites drastically altered the conductivity and electronic states.

Besides, halogen can also doped into O‐site to improve the electrocatalytic activity. Wang and coworkers reported a Cl‐doped LaFeO_3_ used stoichiometric amounts of La(NO_3_)_3_ • 6H_2_O and LaCl_3_ • 7H_2_O as reactants.^[85]^ The Cl‐doped LaFeO_3_ exhibited a smaller Tafel slope than LaFeO_3_, suggesting that OER rates were faster. The authors explained the elevated catalytic activity, in line with the majority of the reported literature, by pointing to the increased electrical conductivity, the emergence of oxygen defects, and the improved Fe‐O covalency.

### 4.2 O‐site vacancies

Oxygen vacancies in perovskites are the most favorable Schottky defects due to low‐formation energies and stable structure of perovskites.[^36^, ^53^, ^78^] They were shown to greatly increase the electrocatalytic activities of perovskites. Additionally, the reaction process may change due to the oxygen vacancies. Therefore, controlling the amount of oxygen vacancies is a good strategy to boost electrocatalytic activity.

**FIGURE 12:**
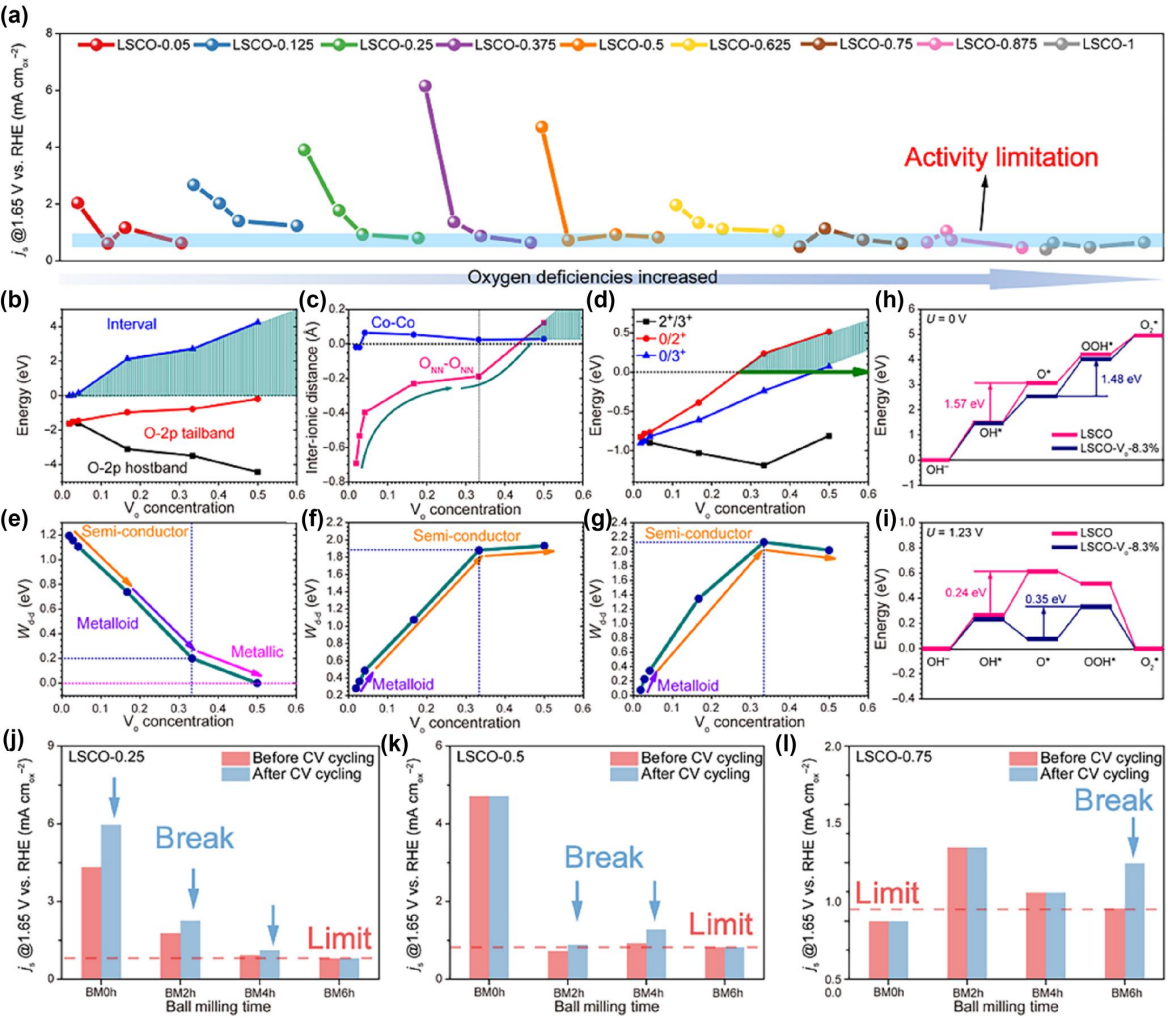
The comprehension of activity variation and mechanism steering caused by oxygen defects. (a) Comparison of the normalized current densities of samples with different concentrations of oxygen defect. The decreased stage of the activity caused by enhanced oxygen deficiency clearly showed the intrinsic OER activity limitation effect. (b) Band offset variation behavior analysis of O 2p hostband, O 2p tailband, and their energetic intervals (the green‐shaded area denotes the large preserved region for locking up the Co^0^ state via pπ compensation). (c) The interionic distance variations of the Co^0^ site near the V_O_ (Co‐Co) and the nearest neighboring O site (O_NN‐NN_). (d) Behaviors of TTLs for the Co site among different charge transitions (0/2^+^), (0/3^+^), and (2^+^/3^+^), respectively. (e–g) The energetic interval between the HOMO and LUMO level (W_d‐d_) of the Co‐3d orbital at the (e) V_O_^0^ state, (f) V_O_^2+^ state, and (g) V_O_^3+^ state. (h, i) The comparison of reaction energy of OER at (h) *U* = 0 and (i) 1.23 V. (j–l) Detailed intrinsic OER activity limitation and mechanism shift optimization analysis of (j) LSCO‐0.25, (k) LSCO‐0.5, and (l) LSCO‐0.75. The separated bars from left to right in each figure represent increased ball milling time and oxygen defects.^[86]^ Copyright (2022) American Association for the Advancement of Science. OER, oxygen evolution reaction.

Recently, Xi and coworkers synthesized a set of La_x_Sr_1−x_CoO_3−δ_ by the sol‐gel method,^[86]^ and material with different content of oxygen vacancies was obtained after ball milling treatment with different time. The oxygen vacancies are observed by HADDF images and annular bright‐field (ABF) images; the concentrations are determined by XAFS detection based on electroneutrality and stoichiometry principles. The authors found three types of mechanism‐shifting models, including AEM‐LOM, AEM‐LOM‐AEM, and LOM‐AEM with different concentrations of oxygen vacancies. By combining the summary of the relationship between oxygen defects and OER activity (Figure 12a) with DFT calculations (Figure 12b–i) for 36 samples, the mechanism of steering and activity changes was further investigated. The Co active sites in the lattice show the concentration locking effect when the oxygen defect concentration increases, and the strong p‐d coupling effect can induce the pπ electron compensation between ions, so that the Co sites can maintain the Co^0^ state for efficient oxygen precipitation reaction. The lattice oxygen activity also shows a volcanic trend with the oxygen defect concentration, which only reaches sufficient activity at the appropriate oxygen defect concentration to promote the OER reaction process. Moreover, the controlled mechanism shift approach (from AEM to LOM) achieved a breakthrough in overcoming the limitation of the intrinsic activity of OER by oxygen defects with a synergistic optimization of catalytic activity and stability (Figure 12j–l). This study provides a new inspiring method to regulate the OER mechanism by the change of oxygen vacancies.

**FIGURE 13:**
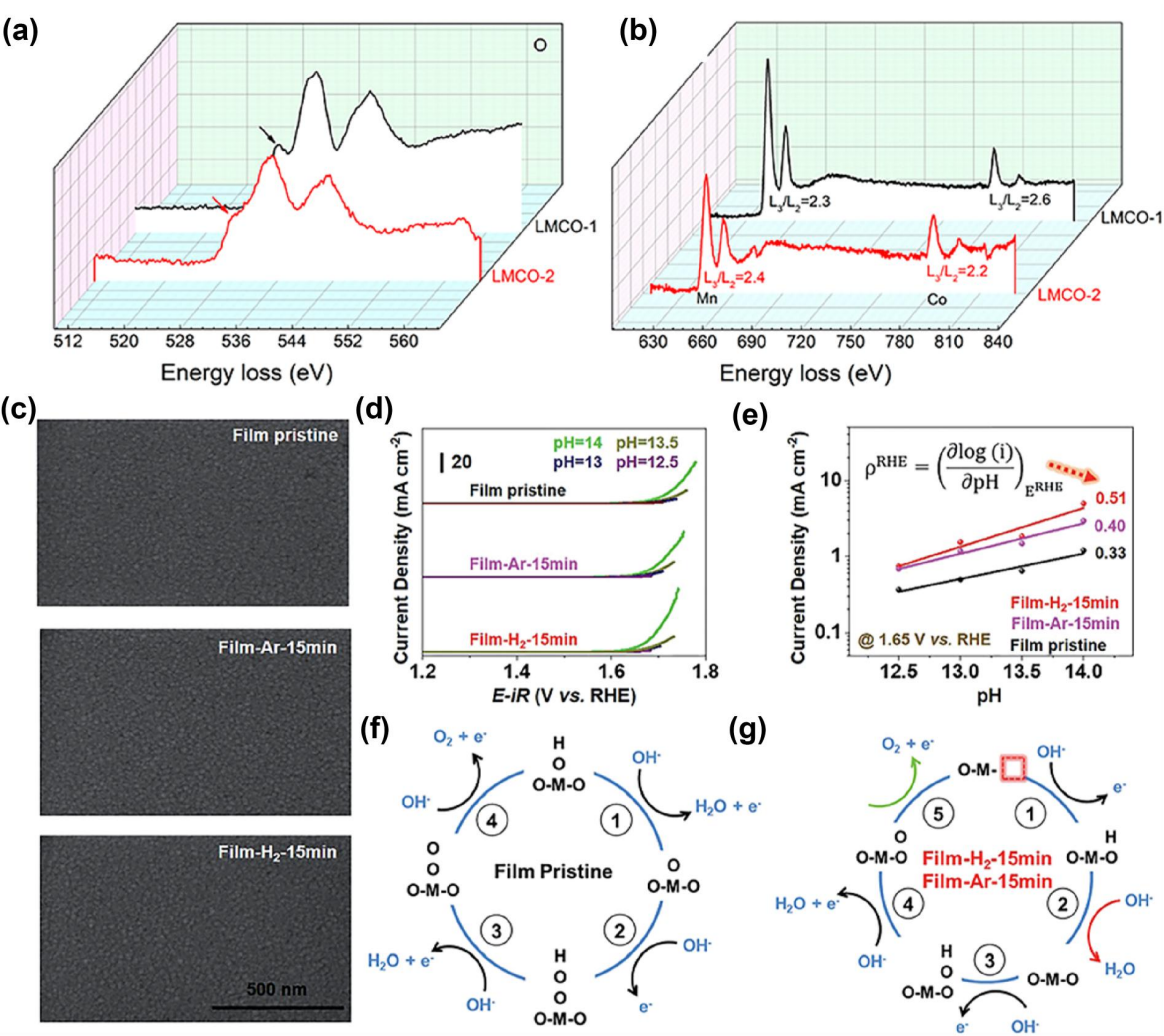
The EELS spectra of LMCO‐1 and LMCO‐2 samples. (a) EELS spectra of the O‐K edge. (b) Overall EELS spectra of Mn‐L and Co‐L edges.^[87]^ Copyright (2019) American Chemical Society. (c) SEM images. (d) Linear sweep voltammetry curves of Film pristine, Film‐Ar‐15 min and Film‐H_2_‐15 min; (e) OER current at 1.65 V versus reversible hydrogen electrode after iR correction plotted in a log scale as a function of pH; (f) and (g) OER mechanism of PBSCF (f) without oxygen defects and (g) with oxygen defects.^[88]^ Copyright (2020) The Royal Society of Chemistry. OER, oxygen evolution reaction.

Numerous investigations used the efficient and highly reproducible hydrogen reduction approach to create oxygen vacancies. In 2019, Sun and coworkers calcined LaMn_0.75_Co_0.25_O_3_ under a reducing H_2_/Ar atmosphere to tune oxygen vacancies in perovskite LaMn_0.75_Co_0.25_O_3−δ_ nanofibers.^[87]^ Local electronic state and coordination environment of the metal ions were characterized by EELS (Figure 13a,b), indicating that the post‐treatment samples had an elevated oxygen vacancy content, elevated Co oxidation state, and opposite Mn oxidation states, which led to a substantial increase in catalytic performance. The samples were also used as a cathode catalyst for zinc‐air batteries, which had a stable cycling performance for 70 h and delivered a high‐power density of 35 mW/cm^2^.

Chen and colleagues treated the PrBa_0.5_Sr_0.5_Co_1.5_Fe_0.5_O_5+δ_(PBSCF) perovskite thin film with Ar and H_2_ plasma to produce oxygen vacancies in the material at varied concentrations (Figure 13c).^[88]^ After plasma treatment, the pH dependence of the OER currents of the films was significantly enhanced, indicating a greater degree of decoupling of the proton and electron transfer processes (Figure 13d,e). The presence of oxygen vacancies also led to a change in the reaction mechanism (Figure 13f,g). The overpotential of the obtained defective PBSCF powder is 90 mV lower than that of commercially available IrO_2_ catalysts at 35 mA/cm^2^, indicating its superior OER performance at high current density.

## 5 CONCLUSION

With the development of various synthetic methods and characterization techniques, a large amount of experimental studies on perovskite oxides have been carried out. Numerous literature reports have demonstrated that catalyst performance enhancement arises from both structure and electron transfer. Obviously, both of them are closely related to the composition of the material. In this review, we have summarized the advancement of research on the regulation of the composition of perovskite oxides in recent years, including A‐site regulation, B‐site regulation, and O‐site regulation. Based on the component regulation of each site, number of e_g_ electrons in the active site, degree of overlap of O 2p‐M 3d, defect content, even the control steps, the degree of reconstruction, and the catalytic mechanism in the reaction process can be reasonably optimized during the regulation of catalysts's compositions. Thus, significant progress has been made in the use of perovskite oxides as electrocatalysts. The catalytic performance of the perovskite oxide catalysts after composition regulation is shown in Table 1. However, there is still a gulf to be crossed between the reported perovskite oxide catalysts and their wide applications.

**TABLE 1:** Catalytic performance comparisons of electrocatalysts obtained through composition regulation and pristine perovskite oxide catalysts.

Composition regulation	Type	Catalysts/pristine materials	Catalytic performance				Electrolyte	Ref.
			OER		ORR^b^			
			η^a^	Taf^b^	E_1/2_ ^c^	Taf.^b^		
A‐site	Ce dopant	Ce_5.6%_‐LaCoO_3_/LaCoO_3_	0.45/0.52	112/125	0.72/0.65	70/78	1 M/0.1 M KOH	[46]
	Ce dopant	La_0.9_Ce_0.1_NiO_3_/LaNiO_3_	0.27/0.46	45/96	‐	‐	1 M KOH	[47]
	Sr dopant	La_0.2_Sr_0.8_FeO_3 −δ_/LaFeO_3_	0.37/0.51	60.1/76.96	‐	‐	0.1 M KOH	[50]
	Sr dopant	La_0.4_Sr_0.6_NiMnO_3_/LaNiMnO_3_	0.367/~0.46^d^	86/107	0.72/0.68	‐	1 M KOH	[51]
	La defects	La_0.95_FeO_3_/LaFeO_3_	0.41/0.51	48/77	‐	82/92	0.1 M KOH	[57]
	Sr defects	LaSr_1.9_Co_1.5_Fe_1.5_O_9−3𝛿_/LaSrCo_1.5_Fe_1.5_O_9−3𝛿_	0.293/0.315^d^	59/70	‐	‐	1 M KOH	
	LaSr excess	(La_0.8_Sr_0.2_)_1.05_MnO_3−δ_/La_0.8_Sr_0.2_MnO_3−δ_	‐	109/140	‐	82/108	0.1 M KOH	[59]
B‐site	Ir dopant	LaNi_0.96_Ir_0.04_O_3_/LaNiO_3_	0.28/0.387	62/93	‐	‐	1 M KOH	[63]
	Fe dopant	LaCo_0.75_Fe_0.25_O_3_/LaCoO_3_	0.31/0.43	58/89	0.84/‐	‐	1 M/0.1 M KOH	[64]
	P dopant	P_500_‐LaFeO_3−δ_/LaFeO_3_	0.465/‐	50/98	0.66/‐	64/72	0.1 M KOH	[69]
	Si dopant	SrCo_0.95_Si_0.05_O_3−δ_/SrCoO_3_	0.417/0.488	66/76	‐	‐	0.1 M KOH	[71]
	CoMn defects	La(Co_0.55_Mn_0.45_)_0.99_O_3−δ_/NrGO	0.787/‐	‐	0.269^f^/‐	‐	0.1 M KOH	[72]
	Ni exsolution	LaNiO_3_/NiO	0.334/0.435	73/93	‐	‐	1 M KOH	[75]
O‐site	N dopant	N‐LaCoO_3_/LaCoO_3_	1.69/1.94^e^	136.6/178.9	‐	51.7/53.5	1 M/0.1 M KOH	[83]
	S dopant	S_5.84%_‐LaCoO_3_/LaCoO_3_	0.364/0.473	126.7/180.9	0.704/0.653	92/111	1 M/0.1 M KOH	[84]
	Cl dopant	LaFeO_2.9‐δ_Cl_0.1_/LaFeO_3_	0.46/0.51	59/69	‐	‐	0.1 M KOH	[85]
	O defects	Ar‐LaMn_0.75_Co_0.25_O_3_/LCMO	~0.45/~0.68	97/143	‐	115/120	0.1 M KOH	[87]
	O defects	15 min H_2_—PrBa_0.5_Sr_0.5_Co_1.5_Fe_0.5_O_5+δ_/PBSCF	0.441/0.488	91.2/101.9	‐	‐	1 M KOH	[88]

Abbreviation: RHE, reversible hydrogen electrode.

^a^ Unless otherwise mentioned, overpotentials in the table are at 10 mA cm^−2^ (V vs. RHE).

^b^ Tafel slope (mv dec^−1^).

^c^ Half‐wave potential (V vs. RHE).

^d^ The overpotentials at 1 mA cm^−2^ (V vs. RHE).

^e^ The overpotentials at 50 mA cm^−2^ (V vs. RHE).

^f^ Half‐wave potential (V vs. Ag/Cl). ∼ Representative estimate based on literature images.

We still believe that perovskite oxides have a great deal of promise to replace noble metal electrocatalysts due to their structural and compositional diversity. In the following, we try to suggest possible directions of efforts to achieve this goal.

First, this paper focuses on the single‐site composition regulation of perovskite oxides. In fact, the simultaneous regulation of dual‐site or even multi‐site has also been shown to be an effective strategy to enhance the catalytic activity. However, compared with the abundant examples of single‐site regulation in both experimental and computational aspects, we lack practical empirical summary for multi‐sites simultaneous regulation. Fortunately, the development and advancement of big data tools, such as high‐throughput DFT computing, machine learning, and artificial intelligence, now give us the opportunity to do so.^[89]^

Second, weak electronic conductivity of perovskite oxides at room temperature is a disadvantage. The effective complex hybridization with other materials to enhance their electrical conductivity may be a good idea. By hybridization, several different components can be introduced at the same time, which also make the tuning of electronic structure and catalytically active components easier. However, it should be noted that the structural instability of chalcogenide oxides, where the A‐site or B‐site elements can be enriched, leached, or dissociated under different experimental conditions, expands the content of nanocomposites while bringing uncertainties to their composition. This also urges us to optimize the synthesis methods and achieve precise control of the nanocomposite structure continuously. Besides, the composites are rich in interfaces, but it is yet unclear how these interfaces function during catalytic reactions, which is a major challenge point to focus on.

Third, structural reconstruction of perovskite oxides can improve their catalytic activity, but there are still many unclear impacts on their stability. Further investigation in the balance between activity and stability of perovskite oxides is still urgently needed to guide the future development of perovskite oxides. Therefore, it is necessary to continue to make a lot of efforts in the in‐situ characterization tools, which make it possible to monitor the synthesis process and the reaction process in real time.

Finally, most of the research studies are limited to laboratory scale, but the gap between basic research and practical applications is far, and the large‐scale application of perovskite oxides is still a great challenge for many researchers. The related technical problems are still to be solved. In summary, combining theoretical calculations with experimental studies for the rational design of perovskite oxide catalysts has a very broad research prospect.

## CONFLICT OF INTEREST STATEMENT

The authors declare no conflict of interests.
